# Didactyl Tracks of Paravian Theropods (Maniraptora) from the ?Middle Jurassic of Africa

**DOI:** 10.1371/journal.pone.0014642

**Published:** 2011-02-14

**Authors:** Alexander Mudroch, Ute Richter, Ulrich Joger, Ralf Kosma, Oumarou Idé, Abdoulaye Maga

**Affiliations:** 1 Initiative of Independent Palaeobiologists, Hannover, Germany; 2 Staatliches Naturhistorisches Museum Braunschweig, Braunschweig, Germany; 3 Institut de Recherches en Sciences Humaines, Université Abdou Moumouni, Niamey, Niger; Raymond M. Alf Museum of Paleontology, United States of America

## Abstract

**Background:**

A new dinosaur tracksite from ?Middle Jurassic sediments of the Irhazer Group on the plains of Agadez (Rep. Niger, northwest Africa) revealed extraordinarily well preserved didactyl tracks of a digitigrade bipedal trackmaker. The distinct morphology of the pes imprints indicates a theropod trackmaker from a paravian maniraptoran closely related to birds.

**Methodology/Principal Findings:**

The early age and the morphological traits of the tracks allow for description of the new ichnotaxon Paravipus didactyloides. A total of 120 tracks are assigned to 5 individual trackways. The ‘medium-sized’ tracks with an average footprint length of 27.5 cm and footprint width of 23.1 cm are deeply imprinted into the track bearing sandstone.

**Conclusions/Significance:**

A comparison with other didactyl tracks gives new insights into the foot morphology of advanced maniraptoran theropods and contributes to knowledge of their evolutionary history. The new ichnotaxon takes an important position in the ichnological fossil record of Gondwana and the mid-Jurassic biota worldwide, because it is among the earliest known records of paravian maniraptorans and of didactyl theropod tracks from Africa.

## Introduction

**Figure 1:**
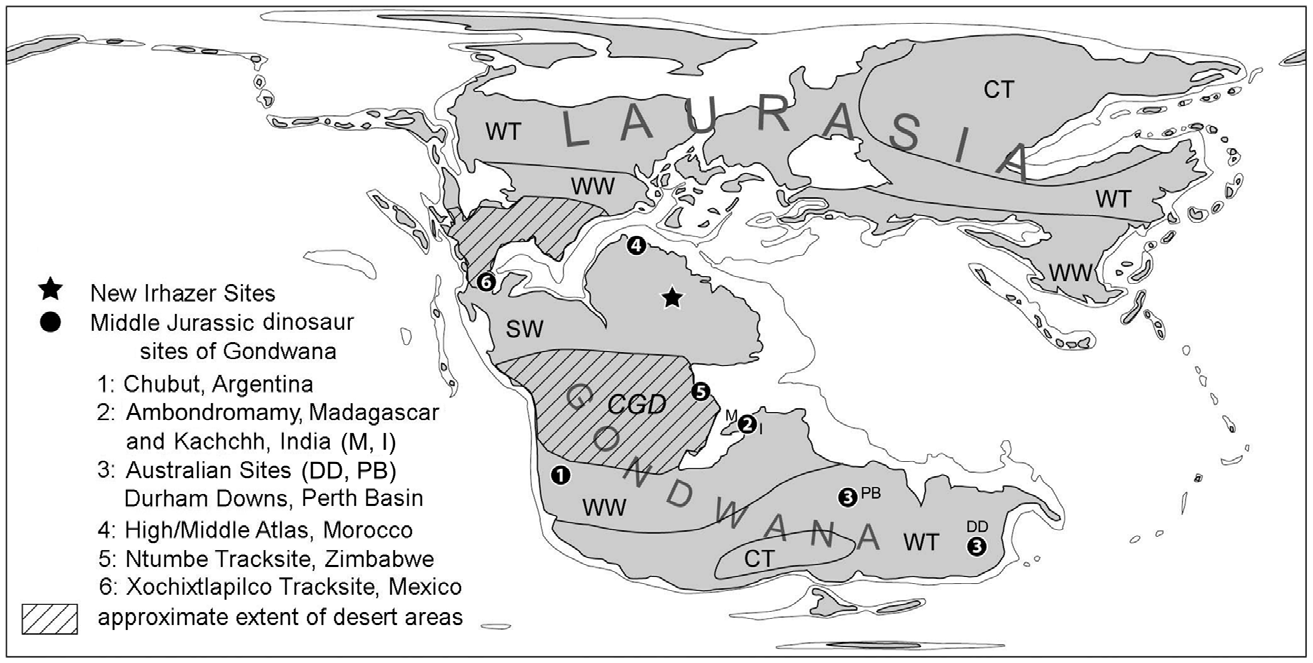
Paleogeographical map of the Middle Jurassic globe with important localities of Gondwana indicated. After Remes et al [22].

The Middle Jurassic (176-161 Ma) was a time of profound paleogeographic change on a global scale [1]. Formerly connected continental plates that once formed the supercontinent Pangaea drifted apart from each other. Spreading of the Proto-Atlantic permanently separated Gondwana and Laurasia with only a narrow Latin American land bridge between North and South America (mostly Mexican terranes), that allowed for occasional faunal exchange until the early Late Jurassic (Fig. 1). Gondwana itself started to break up, producing a large continental seaway in the East that is now part of the Mozambique Channel between Africa and Madagascar. The southern part of Gondwana (Antarctica, Australia, Madagascar, India, and southern South America) was additionally separated from the northern part (North and Central Africa, northern South America) through a natural barrier – the Central Gondwanan Desert (CGD) that covered large parts of Central South America and South Africa [2], [3]. Northern Gondwana was located close to the equator and had a summer-wet climate with high plant productivity and diversity [4]–[7]. Conifers (Gymnosperms) became the dominant group of plants in mid-Jurassic forests, and Cycadales, Bennettitales, and ferns were equally well distributed [8]–[14]. A high diversity of terrestrial vertebrates with dinosaurs as the dominant group is strongly indicated but not much of it is yet recorded.

### Mid-Jurassic dinosaur fauna of Gondwana

Although the dinosaur fossil record of the Middle Jurassic both for Laurasia and Gondwana is poor, available fossils indicate that it was a time of rapid diversification of all existing dinosaur groups [15]. At the beginning of the Late Jurassic almost every lineage of advanced herbivorous and carnivorous dinosaurs was represented [16]. Although the ornithischians were only minor elements in Jurassic terrestrial ecosystems, the Eurypoda (Ankylosauria and Stegosauria) as well as the Cerapoda (basal ornithopods) originated in the Middle Jurassic [17]–[19]. The dominant herbivores of the Jurassic, the sauropodomorph saurischians evolved to larger forms and gave rise to the Neosauropoda [20]–[22]. The carnivores, mainly theropod saurischians, underwent a radiation as well, and there are strong indications that all major clades of tetanuran theropods (Spinosauroidea, Allosauroidea, and Coelurosauria) were already present during or at the end of the Middle Jurassic [23]–[25]. In fact not only basal coelurosaurs like the Tyrannosauroidea [26],[27], but also advanced coelurosaurs like the Eumaniraptora seem to have their roots in the Middle Jurassic [28]–[31]. The mid-Jurassic record of the indicated saurischian diversity is globally poor and strongly biased to the Northern Hemisphere [15], [32]. The main problem of finding fossils of this age is the dating of fossil bearing beds, especially in the Mesozoic continental sediments of Africa.

Despite those circumstances, there are some Gondwanan localities that contribute to the dinosaur record of the Middle Jurassic [15] (Fig. 1). Combined data of bone and track bearing localities clearly enhance our understanding. Some well dated (Bathonian-Callovian) bone and track sites in the High and Middle Atlas region of Morocco (e.g. Demnat, Bin el Ouidane, Isseksi, El Mers) seem to reflect a significant diversity in saurischians comprising several sauropods and at least three types of theropods [32], [33]. Tracks of sauropods and tridactyl theropods of probable tetanuran affinity are reported from the mid-Jurassic Ntumbe tracksite area in Zimbabwe, southern Africa [34], [35]. Farther to the south the Cañadón Asfalto Formation near Chubut, Argentina, has yielded a diverse dinosaurian fauna from the Callovian comprising ornithischians, sauropods and two species of basal tetanurans [15], [36],[37]. Exposures in the Bathonian Isalo III Formation near Ambondromamy, Madagascar, and the Bajocian of the Khadir Island (Kachchh), western India revealed a variety of disarticulated dinosaur material that belongs to ornithischians and saurischians, mainly sauropods but also some theropod teeth of uncertain affinities [15], . Australian findings of mid-Jurassic saurischians (*Rhoetosaurus, Ozraptor*) are reported from Durham Downs, Queensland, and the Colalura Sandstone, Perth Basin [15], [41], but no tracks are coupled with these findings from temperate regions of southern Gondwana. Situated between Laurasia and Gondwana, the Middle Jurassic Xochixtlapilco ichnofauna from the Oaxacan Mixteca in southern Mexico shows apart from ornithopod and sauropod tracks some diversity in theropod tracks with tentative assignments to allosauroids and basal coelurosaurs, but seems to be more closely related to theropod faunas of the Northern Hemisphere [42]. To evaluate this, new bone and track sites like the one herein presented from Gondwana are necessary to better explain the Jurassic dinosaur distribution on southern Continents.

### The new Irhazer sites

**Figure 2:**
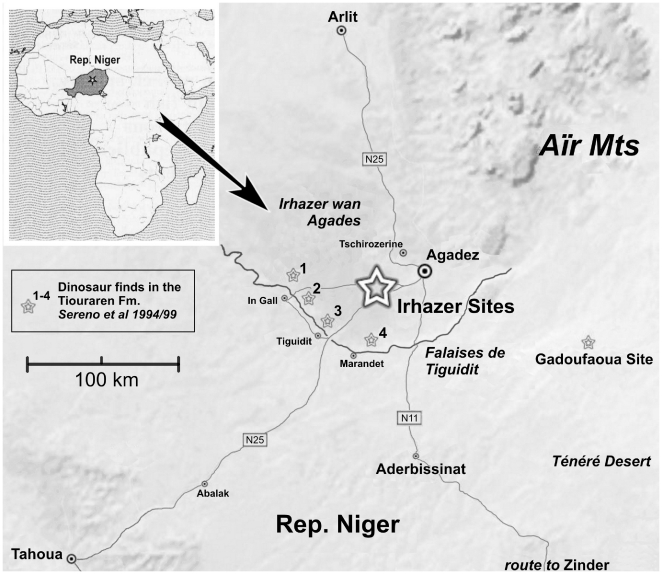
Map of dinosaur localities in the vicinity of Agadez, Rep. Niger. Generated with GoogleEarth MapMaker Utility 2009.

**Figure 3:**
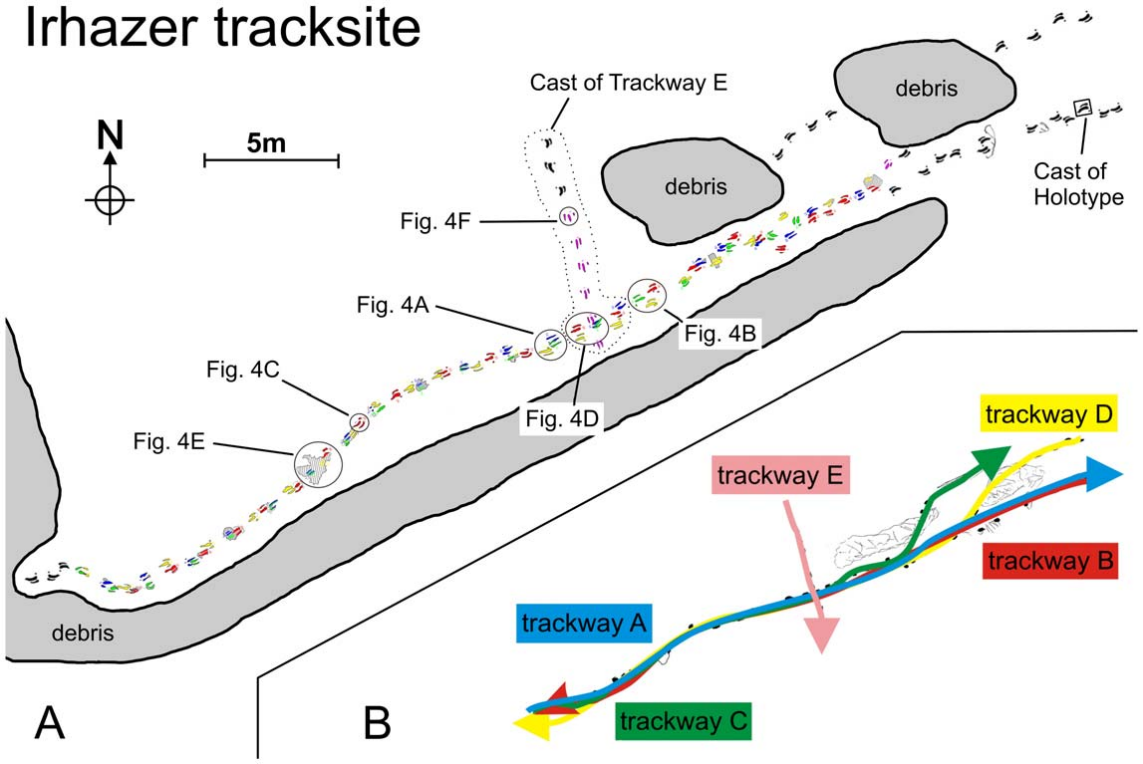
A) Map of the Irhazer theropod tracksite with areas of data coverage indicated. The well documented tracks are numbered and colored. Photos of selected tracks are represented in Figure 4. Casts of the whole trackway E (NMB-1886-Sp) and of the holotype (NMB-1887-Sp) are indicated. B) Trackway pattern below is inferred from individual track recognition and indicated with different colors. Arrows point in the direction of motion for the trackmakers.

**Figure 4:**
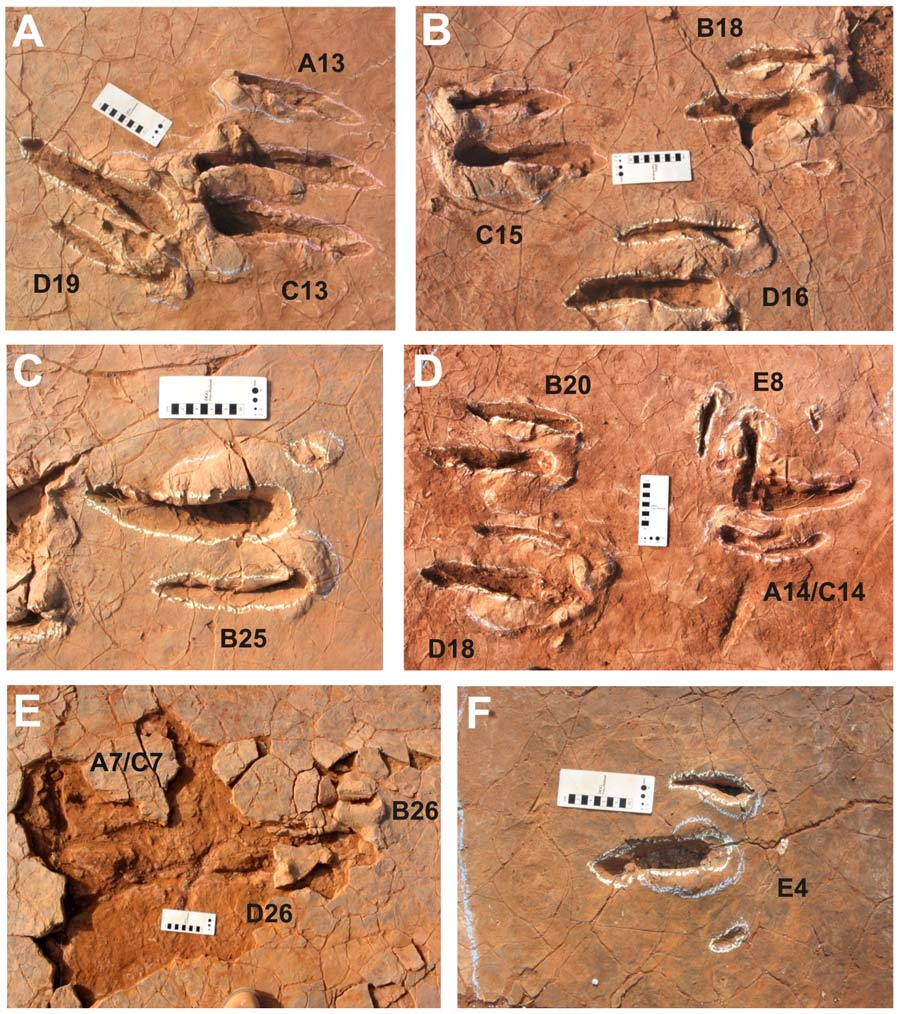
Tracks from different parts of the tracksite area (see **Fig. 3**). Track outlines are highlighted with chalk, and left and right footprint indicated by even and odd numbers. Scale bar equals 10 cm. A) Examples of superimposition and disturbance of tracks A13, C13, and D19. B) Undisturbed tracks B18, C15, and D16 pointing in different directions with prominent sediment bulges around every pedal impression. C) Undisturbed track B25 in an area of larger stride lengths. D) Track A14/C14 to the right results from a superimposition of two nearly identical footprints and is disturbed by track E8 of the crossing trackway; tracks B20 and D18 on the left are undisturbed. E) Undertracks are faintly visible where the original sandstone layer is lost due to erosion. Tracks included are A7, C7, B26, and D26. F) Undisturbed track E4 of crossing trackway.

Mesozoic sediments in the semi-desert plains southwest of Agadez (‘Irhazer wan Agades’, Agadez Region, Rep. of Niger, Fig. 2) revealed a dinosaur bonebed with several excellently preserved specimens of a medium-sized sauropod, recently described as *Spinophorosaurus nigerensis* [22]. Some hundred meters away from the bonebed, 120 exceptionally well preserved didactyl tracks of an unknown bipedal theropod (Fig. 3) and 6 tracks of a medium-sized quadrupedal sauropod were found. The tracks were found in a 3–5 cm thick layer of fine-grained, silty sandstone exposed by erosion from seasonal flooding in an active wadi system. The tracks are preserved as natural moulds in the original track bearing stratum normally filled with the original overlying mudstone of dark red colour (Fig. 4). The overlying mudstone is approximately 30–50 cm thick and still in situ at the southwestern limit of the exposed sandstone surface. Some tracks that were already exposed to the sun are filled up with Quaternary aeolian sand and gravel from the Ténéré desert. Those heavily exposed tracks are in danger of destruction and undertracks are visible (Fig. 4) where the sandstone layer is lost due to weathering. The fine sandstone is situated several centimeters below the siltstone of the bone excavation site in the same lithological unit. It is part of a suite of continental red beds of the Iullemmeden Basin of Niger called the Irhazer Group (Middle to Late Jurassic), in geological maps of North Africa summarized as “Continental Intercalaire”. Lapparent [43] listed two dinosaur bone sites from the Irhazer Group South of Agadez, but they are not identical to the new Irhazer sites.

The lithological unit comprising the track-preserving sandstone layer is called ‘Irhazer Shale’ (Argiles de l'Irhazer) and forms the base of the Irhazer Group. The overlying unit is called the Tiouraren Formation [44], [45] or Dabla Series [46], [47], and it produced several of the new species described from Niger in recent years [48]–[50] (Fig. 2).

The age assignment for the Tiouraren Formation has been the object of serious consideration for a long time with a range of proposed ages from Middle Jurassic to Middle Cretaceous [43], [44], [46], [47], [48]. Direct dating of the sediments and of thin volcanic intercalations therein has given no useful results. A comprehensive study that reviewed all available data for dating concluded that the most likely age for the Tiouraren Formation is late Middle to early Late Jurassic [45]. This estimation was based mainly on paleobiological data and a combination of arguments (e.g. minimum divergence dates for sister group relationships) that indicate at least a Late Jurassic age for the Tiouraren Formation, even if we consider a larger error in the assumptions (e.g. existence of relictual dinosaur faunas). Therefore, the sediments of the unconformable underlying Irhazer Shale must have been deposited between the early Middle Jurassic and the early Late Jurassic. Paleobiological comparison of *Spinophorosaurus nigerensis* from the Irhazer Shale with other basal sauropods from localities worldwide strongly indicates a Middle Jurassic age for these sediments [22]. Both evidences lead to the conclusion that the fossils from the Irhazer sites most likely represent a?Middle Jurassic age.

### Paleoenvironmental situation

The sediments of the Irhazer Group were deposited under fluvio-lacustrine conditions, when the Iullemmeden Basin was part of a great river-valley system that was connected via adjacent basins with the Proto-Atlantic coast of Gondwana [44], [51]. The tracks probably originated on the shore of a small river or lake, because the depth of foot impressions correlating with thick sediment bulges implies a moderate water content of the original unconsolidated sediment (Fig. 4). The fine sandstone that bears the tracks is covered with a pattern of polygonal desiccation cracks. The tracks were imprinted in the bedding plane shortly after the desiccation cracks were formed and disturbed the regular polygonal crack pattern of the sediment. Some of the cracks developed after the track impression. This kind of pattern developed during a continuous desiccation of parts of the moist track-bearing layer. This process of desiccation combined with fast burial by aeolian, fluvial or mixed sediments explains the excellent preservation of the tracks. Additionally microbial mats may have covered the sediment surface as long as conditions were moist enough. A moist or desiccated microbial mat on the sediment surface, forming a thin consolidated layer with water-unsaturated sediment below, would suitably explain the exceptional preservation status of the tracks. These so-called ‘true tracks’ [52] were in most of the cases surrounded by distinct bulges of dislocated sediment (Fig. 4). Displacement rims like those around the pes impressions are reproducible under experimental conditions, as a recent study in microbial mats of modern tidal-flat environments shows [53].

## Results

### The tracksite

We recognized five different trackways (designated A to E) with some 120 didactyl footprints all from the same kind of bipedal runner (Fig. 3). Trackway A comprises 22, trackway B 26, trackway C 19, trackway D 23 and trackway E 9, documented consecutive pes imprints, allowing measurements of pace and stride length and estimations of pace angulation. The lengthy trackways (A–D) show footprints in two main directions with an almost uniform footprint size. Trackway A and C are leading from southwest to northeast, and trackway B and D from northeast to southwest. Impressions in a southwest direction are often disturbed and superimposed by impressions in a northeast direction. This means that trackways B and D are overlapped by trackways A and C, trending in the opposite direction. All imprints are of the same high preservation quality, so that it seems likely that they were emplaced within a very short time span. Apparently the same path that was established by the individuals which generated trackways B and D was used again shortly thereafter by the individuals that generated trackways A and C. Alternatively, the two individuals that left trackways B and D walked back on the same way in the opposite direction and imprinted trackways A and C.

Each trackway is made up of up to 26 alternating individual footprints with variable distances between them, and values from 150–180° for pace angulations. The gait is overall narrow gauge on lengthy trackways, with exceptions when either velocities or direction of movement changes. An average pace length of 117 cm (range: 80–150 cm), the deep pes impressions and the lack of toe drag marks extending anterior to the footprints indicate a rather slow, cautious walking movement of the animals, but with changing velocities of 6–13 km/h.

Trackway E crosses the other trackways in a right angle, trending from northwest to southeast. Individual imprints within this trackway (Fig. 4F) differ from those of the other four trackways in shorter digit lengths and a higher divarication angle between digit III and IV (digit III: average length: 20.25 cm, width: 4.3 cm; digit IV: average length: 14.4 cm, width: 3.36 cm; divarication angle: approximately 15°) but the same overall footprint width of 23.4 cm in average. It seems possible that trackway E was generated considerably later than the other 4 trackways and that the bedding plane lost some of its water content and became more solid under aerial exposition for a few days or weeks. A more solid bedding surface could be the reason for a shorter and stouter appearance of the digit impressions in trackway E, with only two-thirds of every digit impressed in the desiccated bedding surface while the individual walked slowly forward. A pace length of around 75 cm and velocities of around 6 km/h indicate a slow walk for the animal.

The Irhazer tracksite is still partly covered by sediment. Some parts of the exposed track-bearing surface are still buried under piles of debris (Fig. 3). The possible extension of the trackways A, B, C, and D to the southwest direction is not yet excavated.

### Description of tracks

The good state of preservation of many tracks allows for the interpretation of remarkable details of the trackmaker's foot morphology (Fig. 4). Tracks that are superimposed by others gave only partial measurements. All footprints lack traces of the metatarsophalangeal joints and digital nodes. They are typical for theropods with two weight-bearing toes (digit III and IV). The pad impressions of the modified digit II are visible in some of the tracks. They are rarely combined with faint scratch marks (claw marks?).

The average footprint dimensions of all trackways are: length of digit III (‘footprint length’): 27.5 cm (range 18.2–35.6 cm, n = 78), width of digit III: 6.6 cm (range 3.8–9 cm, n = 77); length of digit IV: 20.8 cm (range 10–28 cm, n = 82), width of digit IV: 4 cm (range 2.2–6.6 cm, n = 80); total footprint width: 23.1 cm (range 18.5–28 cm, n = 51).

The divarication angle between digit III and IV ranges from 5° to 15°. Individual pes imprints show a slight rotation with respect to the trackway axis, which means that digit III points nearly parallel to the trackway axis.

### New type ichnogenus and ichnospecies

**Figure 5:**
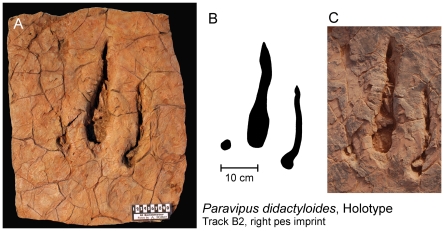
Holotype of new ichnotaxon *Paravipus didactyloides*. Specimen NMB-1887-Sp in the SNMH Braunschweig, Germany. A) Resin cast of the original right footprint made from a silicone mould. B) Silhouette of the original pes imprint. C) Photograph of the original pes imprint in situ. The scale bar is equal to 10 cm for all three images.

***Paravipus didactyloides*** **ichnogen. et ichnosp. nov.**

urn:lsid:zoobank.org:act:AA221E1C-B6E1-4DA4-9824-59EED1C3BAFD

urn:lsid:zoobank.org:act:72051E4F-6CA3-43BB-80FA-2F5EC5380EFD Fig. 5

#### Derivation of name

From the taxon *Paraves* [54], and the Greek *pous*, foot, meaning footprint of a paravian theropod.

From the Greek *di*, two, and *daktylos*, finger or toe, meaning *Paravipus* with a two toed appearance.

#### Holotype

Specimen NMB-1887-Sp in the Natural History Museum Braunschweig, Germany (Staatliches Naturhistorisches Museum Braunschweig, SNHM), resin mould formed from a silicone cast of right footprint No. 2 of trackway B (composed of 32 consecutive footprints).

#### Horizon and type locality

Middle Jurassic, base of the Irhazer-Group (‘Argiles de l'Irhazer’ or Irhazer Shale), plains southwest of Agadez (Irhazer wan Agades), in the Rural Community of Aderbissinat (Thirozerine Dept., Agadez Region, Republic of Niger). GPS coordinates of the tracksite are available on request from the Natural History Museum Braunschweig, Germany (SNHM), for serious research purposes.

#### Diagnosis

Medium sized didactyl pes impressions of a digitigrade bipedal trackmaker exhibiting the following characteristics: slender, separately preserved subparallel impressions of digits III and IV, digit III is slightly longer and wider than digit IV, sharp claw impressions are visible at the ends of the digit impressions and impressions of the metapodium/metatarsus and digital nodes are lacking, both digits revealing a slight laterally convex curvature, digit II is occasionally represented by a small round or oval pad impression preserved slightly behind the posterior/caudal end of digit III marking the proximal portion of digit II. Dimensions of the holotype are: length/width of digit III  = 30.5 cm/7 cm, length/width of digit IV  = 21.5 cm/4 cm, footprint width  = 22 cm. Average footprint dimensions are: footprint length  = 27.5 cm, footprint width  = 23.1 cm. Divarication angle between digit III and IV: 5° to 15°.

## Discussion

### Foot morphology of the trackmaker

A characteristic feature of all members of Deinonychosauria [55] is the unique foot morphology with two larger weight-bearing toes (digit III and IV) and a modified retractable toe (digit II) with a hypertrophied sickle-shaped claw. Footprints of these animals are expected to be didactyl with only a short pad where digit II reaches ground [56]–[58]. Few deinonychosaurian tracks are known, and mainly from the Cretaceous of Asia and North America [57], [59], [60], [61], [62], with one exception from the Tuchengzi Formation of China where the possible age of track bearing sediment ranges from Latest Jurassic to Early Cretaceous [63].

Tracks from the Early Cretaceous of Korea, *Dromaeosauripus hamanensis* [62] resemble the tracks described herein in exhibiting digit III and IV preserved separately, being subparallel to each other and revealing a slight lateral convex curvature, but lacking the impression of digit II. Early Cretaceous tracks documented from Junan County, Shandong Province, China, *Dromaeopodus shandongensis* [57] resemble the tracks described herein only in size (footprint length: 26–28.5 cm), but they differ markedly in that digit III and IV are connected posteriorly which left a distinct metatarsal pad impression. The didactyl tracks of *Velociraptorichnus sichuanensis* from the Early Cretaceous of Emei county [60], [61] and *Menglongipus sinensis* from the Latest Jurassic to Early Cretaceous [63] of Hebei province show significant smaller dimensions: average foot length/width: 11–15 cm/5–8 cm and 5.8–6.7 cm/4–4.7 cm respectively.

Didactyl tracks recently were reported from the Early Jurassic of Gondwana, exposed together with tri- and tetradactyl theropod tracks near Ait Blal, Morocco [64]. The nameless didactyl tracks resemble the tracks described herein only in size (average length of digit III: 27 cm; digit IV: 25 cm), but they differ in that digit III and IV are connected posteriorly by a distinct metatarsal pad impression. Digit III and IV are nearly of the same length and no impression of digit II is preserved. The trackway pattern and the overall resemblance among the di-, tri-, and tetradactyl footprints reported from this Moroccan tracksite suggest the same theropod trackmaker for all the imprints preserved under different substrate conditions, rather than a paravian origin only for the didactyl tracks.

The *Paravipus* trackways represent at least two individuals walking in the same direction. Both individuals provide evidence that digit II was truly modified and not the result of pathology or injury, because of the fact that all the left and right pes imprints of both individuals exhibit a small oval imprint of digit II without any exception along the complete two trackways. That means that only the proximal/posterior part of digit II was involved in weight-bearing during locomotion, a fact already confirmed by previous authors [57], but unique to tracks of Middle to Late Jurassic age. An injured digit II on both the left and right pes of two individuals also seems unlikely. Additionally it is highly unlikely that an animal with two pathologic modified digits II left the same trackway twice exactly along the same place during a short time interval.

The modification of digit II with a hyperextensible joint also discussed for the primitive Late Jurassic basal avian *Archaeopteryx* [65], [66] is an important morphological character for resolving the phylogeny of paravian maniraptorans. The Gondwanan paravian *Rahonavis* [67] that was previously described as an avialan bird is now grouped together with unenlagiine deinonychosaurs [26], [68] mainly on the basis of pedal morphology. The clade Paraves [54] comprises all the direct ancestors of modern birds (Avialae), the deinonychosaurs, a group of carnivorous predators including the clades Dromaeosauridae and Troodontidae with a predominantly Cretaceous fossil record, and all hypothetical members of the stem group. Non-avian paravian theropods, mainly deinonychosaurs, share a number of morphological novelties with modern birds and their relatives, like the reduction/loss of teeth in the whole group of coelurosaurs [69], the widespread presence of feathers in the group of maniraptorans [70], and other anatomical prerequisites for the ability of powered flight among paravians [71], [72] that gives some of these feathered dinosaurs the appearance of true birds. However, the complex character states linked to the possession of a hyperextensible second toe causing functional didactyly are unique to deinonychosaurs [57], even if we concede that some of the modifications on digit II are already present among other advanced maniraptorans.

Aviform tracks from the Mesozoic show quite different morphological features, as they are generally tri- and tetradactyl with wide divarication angles of 80–110° between digit III and IV. The predominantly Jurassic aviform ichnogenus *Trisauropodiscus* from North America and Africa [73] suggests that the available number of weight-bearing toes for Jurassic paravian footprints including birds and deinonychosaurs is two, three, and four, whereas the basal condition among maniraptorans seemed to be only three as for tetanuran theropods in general.

The morphological features of the didactyl pes imprints of the Irhazer tracksite are most congruent with the foot anatomy of articulated deinonychosaur fossils. Several features distinguish the didactyl tracks from all other bipedal dinosaur tracks: the abbreviated impression of digit II indicating a hyperextensible toe, the subequal lengths of the weight bearing digits III and IV, and their sub-parallel orientation and slight inward curvature [57], [62]. Traces of metatarsophalangeal pads at the posterior part of the footprints are not preserved in any *Paravipus* tracks, although the trackmaker's feet were deeply impressed in the moist sediment. That indicates a strictly digitigrade posture of the pes when placed on the track surface. A possible interpretation as swim tracks has to be refuted, as all sedimentological and taphonomical data are clearly contradictory to this.

Only faint undertracks (Fig. 4) of the two weight-bearing digits are visible where the relatively thin (3–5 cm) original layer was removed due to erosion. This leads to the conclusion that body weight of the trackmaker was low in relation to body size. The indicated average hip height of the unknown animal probably ranged between 1.4 and 1.5 meters and total body length between 3 and 4 meters, body size dimensions which resemble very well a medium-sized paravian theropod like *Deinonychus*, although the posture of this animal could have been somewhat different.

### Behavior of the trackmaker

Trackways A, B, C, and D extended along an approximately 1.5 m wide and 40 m long, narrow gauge trackway path (Fig. 3), a rather typical feature of theropod trackways in general since Jurassic times [74], [75]. A temporal succession of trackway origin is indicated by superposition of individual tracks: the earliest tracks belong to trackways B and D, followed after a short while by trackways A and C, leading in the opposite direction and disturbing the imprints of the trackways B and D. Finally, after an indefinite amount of time (probably several days or weeks) trackway E was generated, crossing the other trackways and disturbing some of the tracks. Because of the uniform footprint size across trackways A to D it seems possible that the same two individuals which emplaced the earliest trackways B and D, going in southwest direction, suddenly turned back on their way and went back on the same path in northeast direction, disturbing their own tracks, imprinted minutes or hours before.

Changing velocities of the trackmakers, referable from changing pace and stride lengths depend on physical parameters of the ground and on abrupt changes of moving direction. Velocity estimations based on conventional methods [76] gave speed values of up to 13 km/h for the lengthy trackways (A–D), indicating a rather slow gait, and about 6 km/h on trackway E, indicating even slower movement of the animals. Therefore it is possible that the trackways were emplaced along a physically controlled landscape feature, like a narrow ford leading through an area with water ponds on a muddy flood plain, which forced the animals to find the most secure way to reach the other side of the ancient river system. At one part of the trackway area (Fig. 3) it seems as if the individuals were walking closely together, so that one of them had to give way to the other to avoid a collision. This moment is documented by an abrupt change of pace length and some footprints that were impressed apart from the main trackway axis. Deinonychosaurs have been portrayed as either solitary or gregarious animals, and these tracks could provide evidence of an interaction of at least two individuals walking together with different velocities, a characteristic behavior for agile predators [56], [57], [77], [78].

### Conclusions

Overall features of *Paravipus* tracks imply an unknown Gondwanan member of the paravian clade Deinonychosauria as a possible trackmaker, but there is no record of a medium sized mid-Jurassic deinonychosaur from southern continents yet. *Paravipus* tracks provide evidence that digit II was modified in the trackmaker (and not the result of pathology or injury), with only the posterior part of digit II involved in weight-bearing during locomotion. This is a unique feature in tracks of Middle to Late Jurassic age. The modification of digit II with a hyperextensible joint seemed to have evolved much earlier in the phylogeny of paravian maniraptorans than previously expected.

## Materials and Methods

With permission and assistance of the Archaeological Institute of the University Abdou Moumouni (Institut de Recherches en Sciences Humaines, IRSH), Niamey, Niger, a team of scientists from the SNHM Braunschweig, Germany completed two field seasons, in 2007 and 2008, in the desert plains south of Agadez (Irhazer wan Agades). Adverse circumstances at the remote tracksite allowed only for limited time of data collection, and the surface of the horizontally bedded sandstone layer had to be extensively cleaned of debris in order to see the tracks.

For fast documentation in the field the outlines of all imprints were accentuated with colored chalk, which helped to identify the exact outlines of every single imprint. Three sheets of transparent polyamide plastic each of 3×10 m dimensions and marked with a square meter grid, were then arranged on the lengthy trackways to cover a maximum of tracks with only slight overlap. Outlines of footprints were traced on the sheets with marker pens. Every sheet was oriented with GPS points for later reconfiguration in their original pattern in the lab, where the tracks were studied and their dimensions accurately measured.

Left and right footprints were assigned, numbered, and associated with individual trackways. Tracks were numbered so that even numbers indicate right and uneven left footprints; the letter indicates one of the five trackways. The outlines of footprints were then highlighted with color, transferred to paper sheets, and digitized for further image processing. The scanned image files were digitally enhanced using standard image software and compiled to a digital tracksite map with colored and numbered tracks.

Additionally, plaster and silicone rubber moulds were taken of selected tracks which exhibit characteristic morphological features. Trackway E, with barely disturbed impressions, was completely copied with silicone casts (Fig. 3). All originals of footprints and trackways discussed herein remain in situ at the tracksite. All measurements were taken from the plastic sheets and additionally from silicone casts. One cast was used to generate the holotype discussed below. The silicone casts, the polyester resin moulds made from the casts, and the plastic sheets are housed in the collection of the SNHM Braunschweig (NMB-1886-Sp, NMB-1887-Sp, and NMB-1890-Sp, respectively).

Estimations of velocities (v) for dinosaurs with walking gait (a relative stride length less than 2.0) were calculated with the formula of Alexander as discussed in Thulborn [79]: 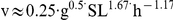



Estimations of the hip height (h) of trackmakers of different size were made with the formulas discussed in Thulborn [80] and Thulborn and Wade [81]:

h≈8.60·FL^0.85^ (for large theropods, FL >25 cm)

Values for pace angulations below 180° were calculated with the formula of Thulborn [78] (PL  =  pace length, SL  =  stride length, a/b =  pace and stride length of left and right pes): 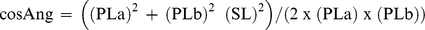



The electronic version of this document though deposited in online digital archives like PubMedCentral and LOCKSS does not represent a published work according to the International Code of Zoological Nomenclature (ICZN), and hence the nomenclatural acts contained in the electronic version are not available under that Code from the electronic edition. Therefore, a separate edition of this document was produced by a method that assures numerous identical and durable copies, and those copies were simultaneously obtainable (from the publication date noted on the first page of this article) for the purpose of providing a public and permanent scientific record, in accordance with Article 8.1 of the Code. The separate print-only edition is available on request from PLoS by sending a request to PLoS ONE, 1160 Battery Street Suite 100, San Francisco, CA 94111, USA along with a check for $10 (to cover printing and postage) payable to “Public Library of Science”.

In addition, this published work and the nomenclatural acts it contains have been registered in ZooBank, the proposed online registration system for the ICZN. The ZooBank LSIDs (Life Science Identifiers) can be resolved and the associated information viewed through any standard web browser by appending the LSID to the prefix “http://zoobank.org/”. The LSID for this publication is: urn:lsid:zoobank.org:pub:158D3BE1-04E5-4219-A91E-A6B33DAA73E4.
